# A case report and systematic literature review: insulin-induced type III hypersensitivity reaction

**DOI:** 10.3389/falgy.2024.1357901

**Published:** 2024-02-21

**Authors:** Rebecca R. Meredith, Pooja Patel, Polly Huang, Chinelo Pamela Onyenekwu, Herleen Rai, Jody Tversky, Santiago Alvarez-Arango

**Affiliations:** ^1^ Division of Hospital Medicine, Department of Medicine, Johns Hopkins University School of Medicine, Baltimore, MD, United States; ^2^ Division of Allergy and Clinical Immunology, Department of Medicine, Johns Hopkins University School of Medicine, Baltimore, MD, United States; ^3^ Division of Transfusion Medicine, Department of Pathology, Johns Hopkins University School of Medicine, Baltimore, MD, United States; ^4^ Division of Clinical Pharmacology, Department of Medicine and Pharmacology and Molecular Science, Johns Hopkins University School of Medicine, Baltimore, MD, United States

**Keywords:** insulin hypersensitivity, immune complex-mediated hypersensitivity, insulin allergy, IgG-mediated hypersensitivity reactions, type III hypersensitivity reaction, insulin resistance, insulin autoantibody

## Abstract

Insulin-induced type III hypersensitivity reactions (HSRs) are exceedingly rare and pose complex diagnostic and management challenges. We describe a case of a 43-year-old woman with type 1 diabetes mellitus (DM), severe insulin resistance, and subcutaneous nodules at injection sites, accompanied by elevated anti-insulin IgG autoantibodies. Treatment involved therapeutic plasma exchange (TPE) and intravenous immunoglobulin (IVIg) as bridge therapy, followed by long-term immunosuppression, which reduced autoantibody levels and improved insulin tolerance. Given the limited treatment guidelines, we conducted a comprehensive literature review, identifying 16 similar cases. Most patients were females with a median age of 36.5 years; 63% had type 1 DM, and 44% had concurrent insulin resistance (56% with elevated autoantibodies). Treatment approaches varied, with glucocorticoids used in 67% of cases. Patients with type 1 DM were less responsive to steroids than those with type 2 DM, and had a more severe course. Of those patients with severe disease necessitating immunosuppression, 66% had poor responses or experienced relapses. The underlying mechanism of insulin-induced type III HSRs remains poorly understood. Immunosuppressive therapy reduces anti-insulin IgG autoantibodies, leading to short-term clinical improvement and improved insulin resistance, emphasizing their crucial role in the condition. However, the long-term efficacy of immunosuppression remains uncertain and necessitates continuous evaluation and further research.

## Introduction

Hypersensitivity reactions (HSRs) to human and analog insulins are rare and can be categorized as immediate or delayed. Immunoglobulin E (IgE) mediated HSRs, known as type I HSRs, generally develop within minutes after injection and can vary from local erythema or a pruritic wheal at the injection site to anaphylaxis. In contrast, delayed T-cell-mediated reactions, type IV HSRs, tend to appear within days as contact dermatitis, marked by eczematous areas. Such reactions are frequently attributed to additives found in insulin formulations (1–3). Infrequently, insulin hypersensitivity can arise from the formation of antigen-antibody immune complexes (ICs), resulting in type III HSRs. These reactions are often characterized by the development of painful subcutaneous nodules, commonly referred to as “Arthus’ reactions”, occurring at the insulin injection sites within 24 h of the subcutaneous injection (2, 4).

We report a unique case involving the concurrent presence of increasing insulin resistance and severely painful subcutaneous nodules at the insulin subcutaneous injection sites in a 43-year-old female with type 1 diabetes mellitus (DM) and high anti-insulin IgG autoantibodies. We hypothesize this presentation to be triggered by the high-titer anti-insulin IgG autoantibodies, resulting in the formation of ICs. These ICs may deposit at the injection sites, causing localized skin reactions as well as potentially inducing insulin resistance through a consumptive process. The pathogenesis of insulin-induced type III HSRs remains poorly understood, and the prevalence of coexisting insulin resistance remains limited, presenting substantial challenges in terms of management.

We conducted a thorough literature review to identify similar cases involving insulin-induced type III HSRs, either in conjunction with or independently of insulin resistance. Within this review, we identified 16 cases with confirmed or suspected type III HSRs, among which 7 exhibited concurrent evidence of insulin resistance. In the cases we reviewed, various immunosuppressive strategies were employed with varying degrees of success. In our case, due to the severity of injection site reactions and insulin resistance, we employed a novel treatment strategy involving therapeutic plasma exchange (TPE) followed by intravenous immunoglobulin (IVIg) as bridge therapy. This was then followed by long-term immunosuppression with rituximab and mycophenolate mofetil (MMF). This treatment resulted in a significant reduction in insulin autoantibody levels, allowing for the successful reintroduction of subcutaneous insulin.

The scarce case reports we identified in our literature review emphasize the exceptional rarity and likely underreporting of insulin-induced type III HSRs with or without insulin resistance. Managing this condition presents a significant challenge due to the absence of clear guidelines. The significant improvement in our patient's reactions and insulin requirements, along with a decrease in anti-insulin IgG autoantibody levels, aligns with the observations in other cases we reviewed. This pattern suggests a potential role for anti-insulin IgG autoantibodies in the pathogenesis of insulin-induced type III HSRs and concurrent insulin resistance. Nonetheless, further research is needed.

## Case

A 43-year-old woman with previously well-controlled type 1 DM, celiac disease, and hypothyroidism presented with a month-long escalation in insulin requirements, uncontrolled hyperglycemia, and painful skin lesions at insulin subcutaneous injection sites. The patient had used an insulin pump for over 30 years without incident. However, one month prior to presentation, her daily insulin requirements began to increase, ultimately doubling from 60 to 120 units/day, with no changes in diet or weight. About a week later, she developed painful skin reactions at injection sites occurring more than 6 h post-injection. Despite multiple attempts with various insulin formulations, her condition failed to improve, resulting in an inability to administer insulin subcutaneously and necessitating hospitalization due to diabetic ketoacidosis (DKA).

**Figure 1:**
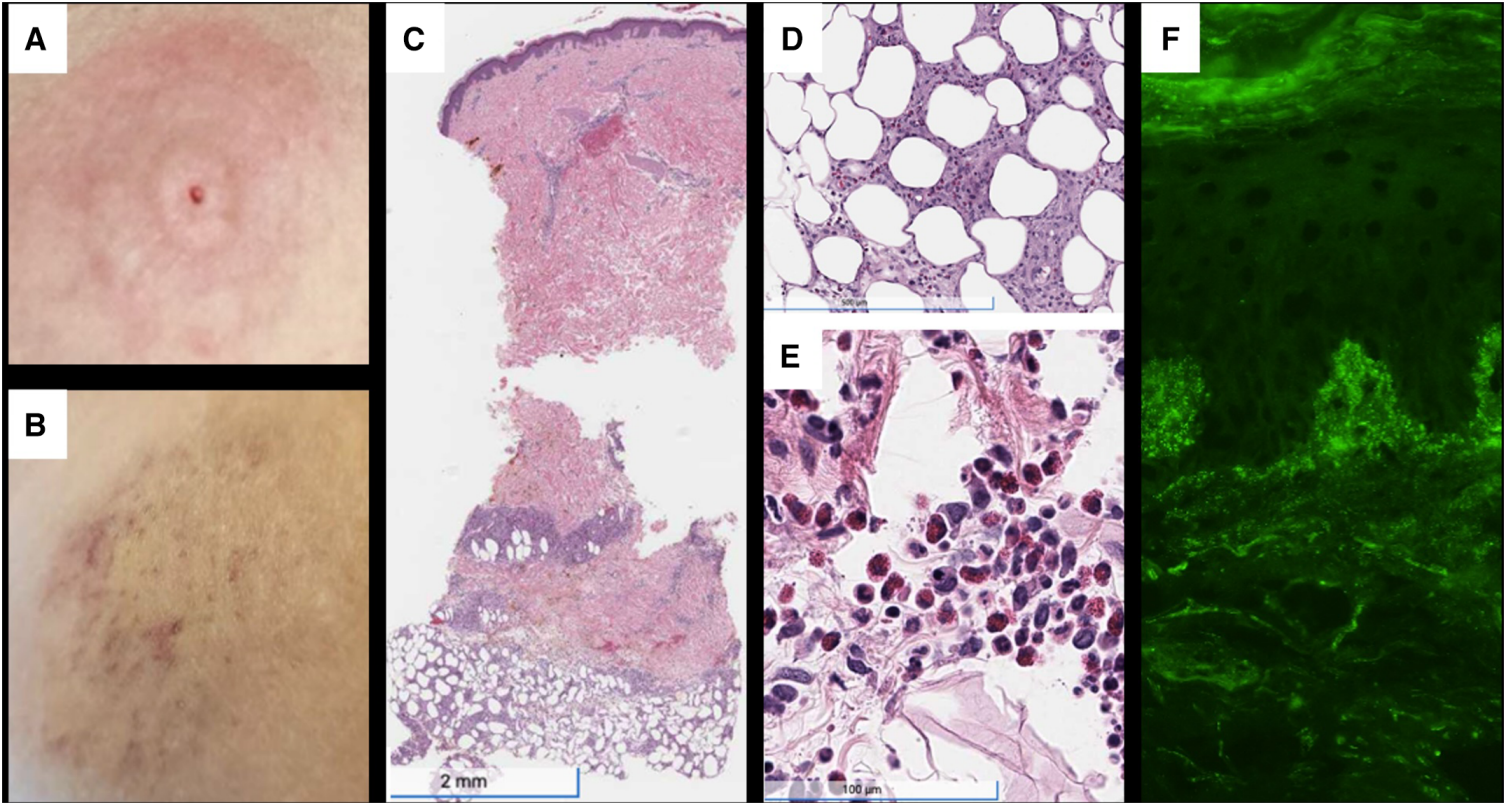
(**A**) Painful, erythematous skin rash at the insulin pump injection site on the upper abdomen, just prior to presentation. (**B**) Area of induration at previous insulin injection site on admission, with evolving erythema, ecchymosis, and petechiae. (**C**) H&E staining demonstrating subcutaneous granulomatous infiltrate with numerous eosinophils. (**D**) 10.5× magnification showing granuloma and eosinophils. (**E**) Superficial and deep perivascular and interstitial infiltrate, including lymphocytes and numerous eosinophils. (**F**) Direct immunofluorescence with C3 deposition in dermal papillae and basement membrane. H&E, hematoxylin and eosin stain.

**Figure 2:**
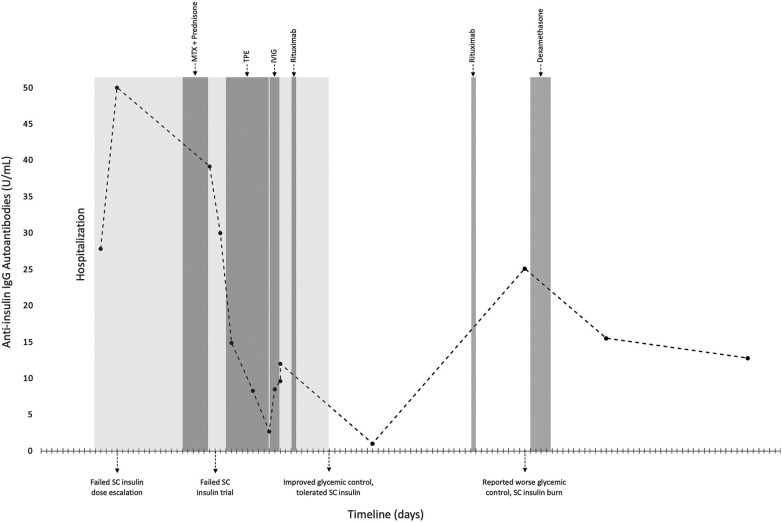
Timeline of the case report of insulin-induced type III HSR and co-existing insulin resistance, illustrating the changes in anti-insulin IgG autoantibody titers, treatment interventions, and the evolution of symptoms over time. HSR, hypersensitivity reaction; IgG, immunoglobulin G; IVIG, intravenous immunoglobulin; MTX, methotrexate; SC, subcutaneous; TPE, therapeutic plasma exchange.

Her physical examination revealed tender, erythematous subcutaneous nodules on the bilateral flanks, lower abdomen, and arms at insulin injection sites (Figures 1A,B). Laboratories revealed glucose >500 mg/dl, bicarbonate 16 mmol/L, anion gap 16 mmol/L, and moderate ketones on urinalysis. Furthermore, laboratories were notable for anti-insulin IgE <0.10 kUA/L and elevated anti-insulin IgG of 27.8 U/ml, subsequently rising to >50 U/ml (Figure 2). Skin biopsies showed mixed dermal infiltrates with prominent lymphocytes and eosinophils and granulomatous subcutaneous infiltrate (Figures 1C–E). Direct immunofluorescence (DIF) showed patchy deposition of C3 in a granular pattern in the superficial dermal papillae with negative IgM and IgG (Figure 1F).

The patient was admitted to the intermediate care unit due to DKA. She responded well to intravenous (IV) insulin therapy without any complications or evidence of an allergic reaction, ruling out a type I IgE-mediated hypersensitivity. Attempts to use various subcutaneous insulin formulations with protamine and metacresol to investigate a potential HSR secondary to additives were unsuccessful. Moreover, a subcutaneous insulin dose escalation protocol was attempted, but painful localized skin reactions persisted at doses ≥4 units.

Given the concern for an IC-mediated HSR, immunosuppression was pursued, initially with methotrexate and prednisone. However, the patient needed escalating IV insulin doses, up to 10 units/h, along with frequent boluses (total daily doses of up to 230–240 units/24 h), to sustain euglycemia. Based on previously reported clinical responses to IVIg and TPE, a combination of both was pursued (5–8). She completed 5 sessions of TPE followed by 2 g/kg IVIg administered over two days. Treatment was complicated by severe headache after completion of IVIg therapy, with cerebrospinal fluid studies concerning for aseptic meningitis, which was successfully treated with a short course of oral glucocorticoids. Following IVIg and TPE, anti-insulin IgG titer significantly declined, from >50 U/ml to 2.7 U/ml (Figure 2). Treatment was then followed with one dose of rituximab, with a planned second dose two weeks later, and the initiation of mycophenolate mofetil (MMF) for long-term immunosuppression. Subcutaneous insulin was slowly reintroduced and effectively titrated to therapeutic doses, leading to the patient's discharge after a 42-day hospitalization.

## Literature review

### Methods and materials

#### Study selection and abstraction

We thoroughly searched the PubMed electronic database, covering articles published from 1959 to 2023. Our search utilized Medical Subject Headings (MESH) Terms, “insulin” and (“allergy” or “hypersensitivity”). We refined the search by filtering for case reports and systematic reviews. Articles without full-text availability were excluded from our study. The initial abstract screening phase involved two independent reviewers (RM and SAA), who assessed the relevance of the identified articles. Subsequently, articles that met our inclusion criteria underwent a thorough full-text screening conducted by three independent reviewers (RM, PP, and SAA). Articles meeting our inclusion criteria proceeded to full-text extraction and in-depth analysis. Lastly, we examined the reference lists of each retrieved article to identify any potential additional cases that met the criteria for our literature review.

We implemented a structured data abstraction form that included country of origin, year of publication, first author, patient demographics, presence of co-morbid autoimmune disease, diabetes type (type 1 vs. type 2), presence of anti-insulin IgG autoantibodies, biopsy results, evidence of insulin resistance, administered medications, utilization of bridge therapy (including TPE or IVIg), immunosuppressive medications administered, use of systemic glucocorticoids, and detailed outcomes and any recorded adverse events (Table 1).

**Table 1:** Summary of included cases.

Reference (year)	Study design	DM type	Elevated anti-insulin IgG (y/n)	Insulin resistance (y/n)	Skin biopsy pathology	Steroid responsive (y/n)	Summary of outcome
Alkhatib et al. (2023) (5)	Case report	Type 1	NR	N	Extravasated red cells, eosinophils, mast cells, and spongiosis.	Y	Response to IVIG not reported. Reported improvement with glucocorticoids but discontinued due to hyperglycemia. Poor response to MTX and rituximab. Ultimately required pancreas transplant
Bayraktar et al. (2009) (6)	Case report	Type 1	NR	N	Not reported	Y	Initial response to glucocorticoids, then relapse. Positive response to TPE but not to MTX and azathioprine. Passed away due to anaphylaxis from TPE while awaiting pancreas transplant
Clarke et al. (2020) (9)	Case report	Type 1	Y	Y	Deep dermal abscess, accompanied by a mixed lobular panniculitis.	N	Poor response to systemic glucocorticoids and azathioprine. Ultimately underwent Islet of Langerhans cell transplant followed by immunosuppression
Darmon et al. (2005) (10)	Case report	Type 1	NR	NR	Not reported	NA	Improved with switching from Determir to Glargine
Edwards et al. (2023) (11)	Case report	Type 1	NR	Y	Septal panniculitis	N	Poor response to glucocorticoids and tacrolimus, MTX, HCQ, and colchicine. Treated with intraperitoneal insulin, with positive response. Developed intraperitoneal infection which was successfully treated with antibiotics.
Friedlander and Bryant (1959) (12)	Case report	NR	Y	Y	Not reported	NA	Positive response to nitrogen mustard, ACTH gel, and tolbutamide
Greenfield et al. (2009) (7)	Case report	Type 2	Y	Y	Not reported	NA	Initial positive response to TPE and MMF but improvement plateaued, so she was treated with monthly IVIG and plasmapheresis (sequence/timing NR). MMF discontinued due to sepsis. Glycemic control worsened. Patient subsequently suffered a fatal cardiac event.
Harvey et al. (2020) (8)	Case report	Type 1	Y	Y	Eosinophilic infiltration	NA	Treated with IVIG and rituximab. Initial positive response, then relapse
Mandrup-Poulson et al. (2002) (13)	Case report	Type 1	Y	NR	Perivascular and interstitial infiltration with neutrophilic and eosinophilic granulocytes and fibrin deposition as well as localized extravasation of erythrocytes in the vascular walls, indicating leucocytoclastic vasculitis.	Y	Positive response to glucocorticoids and azathioprine followed by methotrexate
Muller et al. (2023) (14)	Case report	Type 1	NR	NR	Extensive deep-reaching small vessel vasculitis with the aspect of an urticarial vasculitis	NA	Treatment course not reported
Murray et al. (2017) (15)	Case report	Type 1	NR	N	Findings suggestive of leukocytoclastic vasculitis	N*	Restarted on mercaptopurine and colchicine (which she was on for autoimmune enteropathy), with improved tolerance of subcutaneous insulin. Glucocorticoids were discontinued due to unfavorable risk/benefit profile. Long term being considered for pancreas transplant.
Rachid et al. (2010) (16)	Case report	Type 2	NR	N	Superficial and deep and interstitial infiltration with neutrophils and eosinophils. Vascular wall disruption with erythrocyte extravasation accompanied by fibrinoid necrosis and leukocytoclasia consistent with leukocytoclastic vasculitis	Y	Completed short glucocorticoid course with improvement in symptoms and insulin was discontinued, with resolution of symptoms. Type 2 DM controlled on oral medications.
Silva et al. (1997) (17)	Case report	Type 1	Y^a^	N	NR	Y	Localized reactions improved with prednisone
Takahashi et al. (2022) (18)	Case report	Type 2	Y	Y	NR	Y	Positive response to glucocorticoids. Insulin was discontinued and patient was switched to metformin
Teo et al. (2022) (1)	Case report	Type 2	Y	Y	Dermal edema and a moderate amount of lymphohistiocytic infiltrate admixed with some eosinophils, acute inflammatory yield and fibrinous exudates around the superficial dermal vessels and hair follicles	NA	Localized skin reactions persisted but were “tolerable” so immunosuppression was not pursued
Winocour and Haeney (1986) (19)	Case report	Type 2	Y	N	NR	Y	Positive response to systemic glucocorticoids but discontinued due to GI side effects. Positive response to subcutaneous glucocorticoids

DM, diabetes mellitus, NR, not reported, TPE, therapeutic plasma exchange, MMF, mycophenolate mofetil, MTX, methotrexate, HCQ, hydroxychloroquine.

* As described by the authors, the reported minimal benefit of glucocorticoid treatment did not outweigh the risks, therefore glucocorticoid treatment was discontinued.

^a^ Authors report that the anti-insulin IgG was elevated but reported to be within the normal range for the reported diabetic population.

To ensure the quality and consistency of our data collection process, we employed the Covidence software (Covidence Pty Ltd. in Melbourne, Australia). This software facilitated search result management, application of inclusion criteria, conflict resolution, and review tracking. Additionally, it supported quality assessments and ensured standardized data extraction.

#### Inclusion and exclusion criteria

The inclusion criteria included the presence of subcutaneous nodules and/or indurations within 24 h following insulin administration, the occurrence of painful nodules, cases where continuous subcutaneous insulin dose escalation proved ineffective, as well as the report of type III HSRs, antigen-antibody immune complex-mediated reactions, and/or Arthur's reactions.

Conversely, the studies were excluded if they reported immediate local reactions within less than an hour, urticaria-like lesions without providing a detailed account of subcutaneous nodules or indurations, systemic allergic symptoms (such as generalized urticaria, angioedema, bronchospasm, anaphylaxis), responses to continuous subcutaneous insulin dose escalation, or lesions that occurred more than 24 h after the insulin injection.

## Results

Our search using MESH Terms, “insulin” and (“allergy” or “hypersensitivity”), with a filter for case reports and systematic reviews, initially yielded 367 articles, of which 248 had full text available. After screening the abstracts, we identified 28 articles for further full-text review. Among these, 15 met the inclusion criteria. Furthermore, we identified one additional article through the references of the selected articles, bringing the total to 16 articles that reported suspected or confirmed type III HSR to insulin (Table 1).

Out of the 16 cases we examined, 12 (75%) were female. The median age was 36.5 years, with an interquartile range (IQR) of 32.5 years. Race or ethnicity information was unavailable in 11 out of the 16 cases. Among those with reported race data, there were 3 White patients and 2 Asian patients. Additionally, 10 patients (62.5%) had type 1 DM. Anti-insulin IgG autoantibodies were investigated in 9 (56%) cases and were elevated in all 9. Insulin resistance co-occurred in 7 out of the 16 cases (44%). Lastly, comorbid autoimmune disease was reported in 2 cases, both in patients with type 1 DM (Table 2).

**Table 2:** Demographics, characteristics, and treatment interventions in patients with type 1 vs. Type 2 DM.

Sample characteristics	All^a^ *n* = 16 (%)	Type 1 DM *n* = 10 (%)	Type 2 DM *n* = 5 (%)
Demographics			
Gender			
Male^a^	4 (25)	2 (20)	1
Female	12 (75)	8 (80)	4
Age, median (IQR)	36.5 (32.5)		
Race/Ethnicity			
White	3 (60)^b^		
Asian	2 (40)^b^		
Not reported	11 (69)		
Clinical characteristics			
Comorbid autoimmune disease	2 (12.5)	2 (20)	0
Elevated anti-insulin IgG^a^	9 (56)^c^	4 (40)	4 (80)
Features of insulin resistance^a^	7 (44)	3 (30)	3 (60)
Treatment course^d^	All^a^ *n* = 15 (%)	Type 1 DM *n* = 9 (%)	Type 2 DM *n* = 5 (%)
Glucocorticoids	10 (67)	7 (78)	3 (60)
Plasmapheresis	2 (13)	1 (11)	1 (20)
IVIg	2 (13)	2 (22)	0 (0)
Nonsteroidal immunosuppression^a^	9 (60)	7 (78)	1 (20)

DM, diabetes mellitus, NR, not reported, IVIg, intravenous immunoglobulin.

^a^ There was 1 case in which the type of DM was not reported in a 58 year old male with elevated anti-insulin IgG and features of insulin resistance. Systemic glucocorticoids were not used. There was positive response to nitrogen mustard, ACTH gel, and tolbutamide (12).

^b^ Percentage of those cases in which race is reported (5 cases).

^c^ Includes 1 case in which the anti-insulin IgG was elevated but reported to be within the normal range for the reported diabetic population (17).

^d^ Treatment course was reported for 15 out of 16 cases.

The treatment course was reported for 15 of the cases. Among those, 10 patients (67%) received systemic glucocorticoid treatment. Out of those patients, 4 (36%) had a positive clinical response, defined as clinical improvement following glucocorticoid therapy as described by the authors, and did not require additional immunosuppression. Among the 7 patients with type 1 DM who were treated with systemic glucocorticoids, a positive response was reported in 4 (57%) cases, while a poor response was reported in 3 (43%) cases. Among the 3 patients with type 2 DM treated with systemic glucocorticoids, all 3 (100%) were steroid responsive. Of note, glucocorticoids were discontinued in 3 out of the 10 patients (30%) due to side effects including hyperglycemia and GI symptoms. Among the 15 patients for whom treatment was reported, 9 (60%) had severe diseases that necessitated treatment with steroid-sparing immunosuppression, either alone or in combination with glucocorticoids. Of these patients, 7 (78%) had type 1 DM. In 2 out of the 15 cases (13%), TPE was employed, with positive short-term outcomes in both instances, and 2 (13%) patients received IVIg, with mixed short-term outcomes (Tables 1, 2).

Among the 9 patients treated with immunosuppression, 6 (67%) either had a poor clinical response or eventually experienced a relapse of their condition after an initial positive clinical response. Notable outcomes also included one patient who showed no initial positive response to immunosuppression and required a pancreas transplant, another patient who did not respond initially and underwent an Islet of Langerhans cell transplant, one patient who died from anaphylaxis during TPE while awaiting a pancreas transplant, and another who experienced a relapse in glycemic control after discontinuation of immunosuppressive therapy due to sepsis, ultimately resulting in poor glycemic control and a fatal cardiac event (Table 1).

Other outcomes include one patient who experienced symptom remission upon switching to a different insulin formulation, one patient who had remission with the use of subcutaneous glucocorticoids alone, two patients in whom insulin was discontinued in favor of oral hypoglycemics, and one patient who did not receive treatment because the injection site reactions were considered tolerable. Notably, in all of those instances, the patients had type 2 DM (Table 1).

## Discussion

Insulin-induced type III HSRs are exceptionally rare and pose distinctive diagnostic and management challenges. Key indicators involve the development of a delayed-onset, non-urticarial, and painful rash at insulin injection sites. While definitive tests are not available, the presence of elevated anti-insulin IgG autoantibodies and skin biopsy findings, such as subcutaneous granulomas and DIF showing the presence of complement or IgG deposits, should prompt suspicion. Other biopsy findings that may be associated with insulin-induced type III HSRs include red cell extravasation (indicating leukocytoclastic vasculitis) and panniculitis, although it is important to note that several cases reported biopsy findings of nonspecific inflammatory infiltrates (Table 1). Moreover, DIF sensitivity varies among diseases, with higher rates seen in conditions like vesiculobullous diseases and small-vessel vasculitis (20). In certain cases, like ours, insulin-induced type III HSRs coincide with autoimmune insulin resistance, adding complexity to management, especially for type 1 DM patients. Among these patients, many prove to be resistant to steroids, and while some exhibit partial acute responses to bridge therapies involving TPE and/or IVIg, the majority do not respond to immunosuppression or experience relapses after an initial positive response. Long-term management of these patients necessitates multimodal immunosuppression strategies. However, the long-term effectiveness of this treatment approach remains to be determined and requires ongoing follow-up assessment.

Since the introduction of purified insulins and human insulin, the prevalence of anti-insulin autoantibodies in patients previously treated with insulin has declined; however, prevalence remains high. Wredling et al. found that among individuals with prior insulin treatment, up to 78% had insulin autoantibodies, particularly prevalent in type 1 DM patients and those with prolonged insulin use (21). Although rarely clinically significant, anti-insulin IgG autoantibodies can bind to exogenous insulin and form ICs that can deposit in various tissues, triggering the classical complement pathway and causing inflammation. When this process occurs in the skin, it presents with granulomatous lesions and painful nodules at the insulin injection sites (22, 23). Furthermore, immunologic insulin resistance may develop due to the formation of ICs; however, its exact prevalence remains unknown, with only isolated case reports available (22, 24). Berson et al. demonstrated that in individuals with suspected immunologic insulin resistance caused by anti-insulin autoantibodies, these antibodies bound to insulin, forming ICs that neutralized insulin effects (25). In several cases, including ours, immunosuppressive therapy reduced anti-insulin IgG autoantibodies, leading to clinical improvement and a decrease in insulin resistance, highlighting the significance of these autoantibodies.

Treatment for insulin-induced type III HSRs is challenging due to their rarity and reliance on individual case reports, resulting in varying approaches and outcomes. This complexity is amplified in patients with type 1 DM because of their insulin dependence. Glucocorticoids, although offering temporary relief, often prove ineffective in preventing skin reactions and may worsen hyperglycemia. Notably, most cases, including ours, involved individuals with type 1 DM, and a significant portion showed a weaker response to glucocorticoids, requiring alternative forms of immunosuppression compared to those with type 2 DM. Additionally, 57% of the patients with type 1 DM did not respond to immunosuppression and among those who did respond, most experienced relapses after an initial positive response (Table 2). Interestingly, there were 3 cases, including ours, with reported comorbid autoimmune disease, all occurring in patients with type 1 DM. The increased occurrence of type III HSRs in individuals with type 1 DM may be linked to their heightened susceptibility to other autoimmune conditions and autoantibody formation, highlighting the importance of further research in this field.

Both TPE and IVIg have been utilized in the treatment of insulin-induced type III HSRs. TPE was effective in 3 cases, including ours, while IVIg's effectiveness varied, with an initial positive response reported in one case (4–8). Immunosuppressive agents targeting B and T cells, such as rituximab, methotrexate, azathioprine, and MMF, have yielded varying effectiveness results (Table 1). In our case, due to the severity of the clinical course, with ongoing high IV insulin requirements, we adopted a novel approach that combined TPE and IVIg, which resulted in a rapid improvement in clinical symptoms and subcutaneous insulin tolerance, as well as a reduction in anti-insulin IgG autoantibody titers. Compared to using TPE alone, the effectiveness of combining TPE and IVIg on the clinical course and auto-antibody levels remains uncertain. We withheld reintroducing subcutaneous insulin during TPE due to persistently high IV insulin requirements to avoid further burdening the system until tolerance was assured.

Determining whether the observed effects resulted from TPE, IVIg, or their combination is inconclusive. Mechanistically, the reduction in autoantibody titers attributed to TPE is a plausible hypothesis, while the precise impact of IVIg on autoantibody titers is less evident. TPE operates by eliminating intravascular antibodies, whereas the immunomodulatory actions of IVIg include interference with the autoantibody-antigen complex, disruption of complement activation, and modulation of T and B cell activation (26). Nevertheless, what we can confirm is that about three days after IVIg treatment was completed, the patient's IV insulin requirements significantly decreased, facilitating the successful reintroduction of subcutaneous insulin and leading to her discharge from the hospital. Subsequent long-term immunosuppression with rituximab and MMF initially maintained a low anti-insulin IgG level (0.3 U/ml) with optimal glycemic control and sustained subcutaneous insulin tolerance at short-term hospital follow-up.

Upon discharge, her allergy/immunology team planned a second Rituximab infusion two weeks after the first, but it was delayed due to insurance issues. She received the second dose 32 days after the first infusion. Around the same time, insulin requirements increased again, accompanied by burning sensations at injection sites and an elevated anti-insulin IgG level to 25.1 U/ml. An acute intervention involving a four-day steroid pulse regimen led to a temporary reduction of IgG levels to 12.8 U/ml and an improvement in symptoms. Since then, she has received two additional doses of Rituximab. While localized reactions have significantly decreased in frequency, they continue to occur intermittently, up to 2–3 times per week. Insulin requirements have decreased by approximately 45% since hospital discharge, indicating some improvement in insulin resistance with immunosuppression, and she has not re-required hospitalization. However, due to persistent localized reactions and insulin requirements still above baseline, introducing an alternative immunosuppressive medication into the treatment plan and considering a potential pancreatic transplant are actively being explored. Our case and literature review highlight the complexities in managing insulin-induced type III HSRs, especially when they co-occur with insulin resistance, particularly in patients with type 1 DM. This emphasizes the critical need for research in this field.

## Conclusion

Delayed non-urticarial skin reactions to subcutaneous insulin, accompanied by an elevated anti-insulin IgG autoantibody titer, should raise suspicion of insulin-induced type III HSR. In rare instances, coexisting insulin resistance may result from anti-insulin IgG autoantibodies. Managing this condition poses significant challenges due to its exceptional rarity, likely underreporting, and the absence of clear guidelines. There is a high rate of treatment failure and relapse, especially among those with type 1 DM. Treatment should involve bridge therapy with TPE and/or IVIg to promptly lower autoantibody levels, followed by systemic multimodal immunosuppression. Long-term effectiveness remains uncertain and requires ongoing assessment.

## Data Availability

The original contributions presented in the study are included in the article/Supplementary Material, further inquiries can be directed to the corresponding author.
